# Mask R-CNN and OBIA Fusion Improves the Segmentation of Scattered Vegetation in Very High-Resolution Optical Sensors

**DOI:** 10.3390/s21010320

**Published:** 2021-01-05

**Authors:** Emilio Guirado, Javier Blanco-Sacristán, Emilio Rodríguez-Caballero, Siham Tabik, Domingo Alcaraz-Segura, Jaime Martínez-Valderrama, Javier Cabello

**Affiliations:** 1 Multidisciplinary Institute for Environment Studies “Ramon Margalef” University of Alicante, Edificio Nuevos Institutos, Carretera de San Vicente del Raspeig s/n San Vicente del Raspeig, 03690 Alicante, Spain; jaime.mv@ua.es; 2 Andalusian Center for Assessment and monitoring of global change (CAESCG), University of Almeria, 04120 Almeria, Spain; jcabello@ual.es; 3 College of Engineering, Mathematics and Physical Sciences, University of Exeter, Penryn Campus, Cornwall TR10 9EZ, UK; jb1230@exeter.ac.uk; 4 Agronomy Department, University of Almeria, 04120 Almeria, Spain; e.rodriguez-caballer@mpic.de; 5 Centro de Investigación de Colecciones Científicas de la Universidad de Almería (CECOUAL), 04120 Almeria, Spain; 6 Department of Computer Science and Artificial Intelligence, University of Granada, 18071 Granada, Spain; siham@ugr.es; 7 Department of Botany, Faculty of Science, University of Granada, 18071 Granada, Spain; dalcaraz@ugr.es; 8 iEcolab, Inter-University Institute for Earth System Research, University of Granada, 18006 Granada, Spain; 9 Department of Biology and Geology, University of Almeria, 04120 Almeria, Spain

**Keywords:** deep-learning, fusion, mask R-CNN, object-based, optical sensors, scattered vegetation, very high-resolution

## Abstract

Vegetation generally appears scattered in drylands. Its structure, composition and spatial patterns are key controls of biotic interactions, water, and nutrient cycles. Applying segmentation methods to very high-resolution images for monitoring changes in vegetation cover can provide relevant information for dryland conservation ecology. For this reason, improving segmentation methods and understanding the effect of spatial resolution on segmentation results is key to improve dryland vegetation monitoring. We explored and analyzed the accuracy of Object-Based Image Analysis (OBIA) and Mask Region-based Convolutional Neural Networks (Mask R-CNN) and the fusion of both methods in the segmentation of scattered vegetation in a dryland ecosystem. As a case study, we mapped *Ziziphus lotus*, the dominant shrub of a habitat of conservation priority in one of the driest areas of Europe. Our results show for the first time that the fusion of the results from OBIA and Mask R-CNN increases the accuracy of the segmentation of scattered shrubs up to 25% compared to both methods separately. Hence, by fusing OBIA and Mask R-CNNs on very high-resolution images, the improved segmentation accuracy of vegetation mapping would lead to more precise and sensitive monitoring of changes in biodiversity and ecosystem services in drylands.

## 1. Introduction

Dryland biomes cover ~47% of the Earth’s surface [1]. In these environments, vegetation appears scattered [2] and its structure, composition and spatial patterns are key indicators of biotic interactions [3], regulation of water, and nutrient cycles at landscape level [4]. Changes in the cover and spatial patterns of dryland vegetation occur in response to land degradation processes [5]. Hence, methods to identify and characterize vegetation patches and their structural characteristics can improve our ability to understand dryland functioning and to assess desertification risk [5,6,7,8]. Progress has been made using remote sensing tools in this regard (e.g., quantification of dryland vegetation structure at landscape scale [9], monitoring vegetation trends [10], spatial patterns identifying ecosystem multifunctionality [11], characterizing flood dynamics [12], among many others). However, the improvement in the accuracy of vegetation cover measurement is still being studied to obtain maximum performance from data and technology. Estimating and monitoring changes in vegetation cover through remote sensing is key for dryland ecology and conservation [6]. Both historical temporal and spatial data are the base for remote sensing studies to identify the functioning and structure of vegetation [13,14].

The analysis of very high-resolution images to detect and measure vegetation cover and its spatial arrangement across the landscape starts typically by segmenting the objects to be identified in the images [7]. Object-Based Image Analysis (OBIA) [15] and Mask Region-based Convolutional Neural Networks (Mask R-CNN) [16] are among the most used and state-of-the-art segmentation methods. Though they provide a similar product, both methods rely on very different approaches. OBIA combines spectral information from each pixel with its spatial context [17,18]. Similar pixels are then grouped in homogenous objects that are used as the basis for further classification. Mask R-CNN, on the other hand, a type of artificial intelligence whose functioning is inspired by the human brain provides transferable models between zones and semantic segmentation with unprecedented accuracy [19,20]. Besides, fusion has recently been used to improve spectral, spatial, and temporal resolution from remote sensing images [21,22,23]. However, the fusion of methods for vegetation mapping has not been evaluated.

Remote sensing studies based on very high-resolution images have increased in the last years (e.g., [24,25,26,27]), partly because of the availability of Google Earth images worldwide [28,29,30] and the popularization of unmanned aerial vehicles (UAV). Although these images have shown a high potential for vegetation mapping and monitoring [31,32,33], two main problems arise when they are used. First, higher spatial resolution increases the spectral heterogeneity among and within vegetation types, resulting in a salt and pepper effect in their identification that does not correctly characterize the actual surface [34]. Second, the processing time of very high-resolution images and the computational power required is larger than in the case of low-resolution images [35]. Under these conditions, traditional pixel-based analysis has proved to be less accurate than OBIA or Mask R-CNN for scattered vegetation mapping [15,36]. There are many applications for OBIA [37,38,39] and deep learning segmentation methods [40,41]. For example, mapping greenhouses [42], monitoring disturbances affecting vegetation cover [5], or counting scattered trees in Sahel and Sahara [43]. These methods have been compared with excellent results in both segmenting and detecting tree cover and scattered vegetation [7,44,45]. However, greater precision is always advisable in problems of very high sensitivity [46]. Despite methodological advances, selecting the appropriate image source is key to produce accurate segmentations of objects, like in vegetation maps [47,48], and there is no answer to the question of which image or method to choose for segmenting objects. Understanding how the spatial resolution of the imagery used affects these segmentation methods or the fusing of both is key for their correct application to obtain better accuracy in object segmentation in vegetation mapping in drylands.

To evaluate which is the most accurate method between OBIA and Mask R-CNN to segment scattered vegetation in drylands and to understand the effect of the spatial resolution of the images used in this process, we assessed the accuracy of these two methods in the segmentation of scattered dryland shrubs and compared how final accuracy varies as does spatial resolution. We also check the accuracy of the fusion of both methods.

This work is organized as follows. Section 2 describes the study area, the dataset used, and the methodologies tested. Section 3 describes the experiments addressed to assess the accuracies of the methods used. The experimental results and discussion are presented in Section 4, and conclusions are given in Section 5.

## 2. Materials and Methods

### 2.1. Study Area

We focused on the community of *Ziziphus lotus* shrubs, an ecosystem of priority conservation interest at European level (habitat 5220* of Directive 92/43/EEC), located in Cabo de Gata-Níjar Natural Park (36°49′43′′ N, 2°17′30′′ W, SE Spain), one of the driest areas of continental Europe. This type of vegetation is scarce and patchy, which appears surrounded by a matrix of bare soil and small shrubs (e.g., *Launea arborescens*, *Lygeum spartum* and *Thymus hyemalis*). *Z. lotus* is a facultative phreatophyte [49] and forms large hemispherical canopies (1–3 m tall) that constitute fertility islands where many other species of plants and animals live [50]. These shrubs are long-lived species contributing to the formation of geomorphological structures, called nebkhas [51], that protect from the intense wind erosion activity that characterizes the area, thereby retaining soil, nutrients, and moisture.

### 2.2. Dataset

The data set consisted of two plots (Plot 1 and Plot 2) with 3 images of different spatial resolution in each one. The plots had an area of 250 × 250 m with scattered *Z. lotus* shrubs. The images were obtained from optical remote sensors in the visible spectral range, Red, Green and Blue bands (RGB) and spatial resolutions of < 1 m/pixel:

- A 0.5 × 0.5 m spatial resolution RGB image obtained from Google Earth [52].
- A 0.1 × 0.1 m spatial resolution image acquired using an RGB camera sensor of 50 megapixels (Hasselblad H4D) equipped with a 50 mm lens and charge-coupled device (CCD) sensor of 8176 pixels × 6132 pixels mounted on a helicopter with a flight height of 550 m.
- A 0.03 × 0.03 m spatial resolution image acquired using a 4K pixels resolution RGB camera sensor on a professional UAV Phantom 4 UAV (DJI, Shenzhen, China) and with a flight height of 40 m.

### 2.3. OBIA

OBIA-based segmentation is a method of image analysis that divides the image into homogeneous objects of interest (i.e., groups of pixels also called segments) based on similarities of shape, spectral information, and contextual information [17]. It identifies homogeneous and discrete image objects by setting an optimal combination of values for three parameters (i.e., Scale, Shape, and Compactness) related to their spectral and spatial variability. There are no unique values for any of these parameters, and their final combination always depends on the object of interest, so finding this optimal combination represents a challenge due to the vast number of possible combinations. First, it is necessary to establish an appropriate Scale level depending on the size of the object studied in the image [43]; for example, low Scale values for small shrubs and high Scale values for large shrubs [44,45]. Recent advances have been oriented in developing techniques (e.g., [53,54,55,56,57,58,59]) and algorithms (e.g., [60,61,62,63]) to automatically find the optimal value of the Scale parameter [64], which is the most important for determining the size of the segmented objects [65,66]. The Shape and the Compactness parameters must be configured too. While high values of the Shape parameter prioritize the shape over the colour, high values of the Compactness parameter prioritize compactness of the objects over the smoothness of their edges [67].

### 2.4. Mask R-CNN

In this problem of locating and delimiting the edges of dispersed shrubs, we used a computer vision technique named instance segmentation [68]. Such technique infers a label for each pixel considering other nearby objects, thus including the boundaries of the object. We used Mask R-CNN segmentation model [16], which extends Faster R-CNN detection model [16] and provides three outputs for each object: (i) a class label, (ii) a bounding box that delimits the object and (iii) a mask which delimits the pixels that constitute each object. In the binary problem addressed in this work, Mask R-CNN generates for each predicted object instance a binary mask (values of 0 and 1), where values of 1 indicate a *Z. lotus* pixel and 0 indicates a bare soil pixel. 

Mask R-CNN relies on a classification model for the task of feature extraction. In this work, we used ResNet 101 [69] to extract increasingly higher-level characteristics from the lowest to the deepest layer levels.

The learning process of Mask R-CNN is influenced by the number of epochs, which is the number of times the network goes through the training phase, and by other optimizations such as transfer-learning or data-augmentation (see Section 3.2). Finally, the 1024 × 1024 × 3 band image input is converted to 32 × 32 × 2048 to represent objects at different scales via the characteristic network pyramid.

### 2.5. Segmentation Accuracy Assessment

The accuracy of the segmentation task in this work was assessed with respect to ground truth by using the Euclidean Distance v.2 (ED2; [70]), which evaluates the geometric and arithmetic discrepancy between reference polygons and the segments obtained during the segmentation process. Both types of discrepancy need to be assessed. As reference polygons, we used the perimeter of 60 *Z. lotus* shrubs measured with photo-interpretation in all images by a technical expert. We estimated the geometric discrepancy by the “Potential Segmentation Error” (PSE; Equation (1)), defined as the ratio of the total area of each segment obtained in the segmentation that falls outside the reference segment and the total area of reference polygons as:

(1) $$PSE=\frac{\Sigma \left|s_{i}-r_{k}\right|}{\Sigma \left|r_{k}\right|}$$

where PSE is the “Potential Segmentation Error”, r_k_ is the area of the reference polygon and si is the overestimated area of the segment obtained during the segmentation. A value of 0 indicates that segments obtained from the segmentation fit well into the reference polygons. Conversely, larger values indicate a discrepancy between reference polygons and the segments.

Although the geometric relation is necessary, it is not enough to describe the discrepancies between the segments obtained during the segmentation process and the corresponding reference polygons. To solve such problem the ED2 index includes an additional factor, the “Number-of-Segmentation Ratio” (NSR), that evaluates the arithmetic discrepancy between the reference polygons and the generated segments (Equation (2)):

(2) $$NSR=\frac{abs\left(m-v\right)}{m}$$

where NSR is the arithmetic discrepancy between the polygons of the resulting segmentation and the reference polygons and abs is the absolute value of the difference of the number of reference polygons, m, and the number of segments obtained, v.

Thus, the ED2 can be defined as the joint effect of geometric and arithmetic differences (Equation (3)), estimated from PSE and NSR, respectively, as:

(3) $$ED2=\sqrt{{\left(PSE\right)}^{2}+{\left(NSR\right)}^{2}}$$

where ED2 is Euclidean Distance v.2, PSE is Potential Segmentation Error, and NSR is Number-of-Segmentation Ratio. According to Liu et al. [70], values of ED2 close to 0 indicate good arithmetic and geometric coincidence, while high values indicate a mismatch between them.

## 3. Experiments

**Figure 1:**
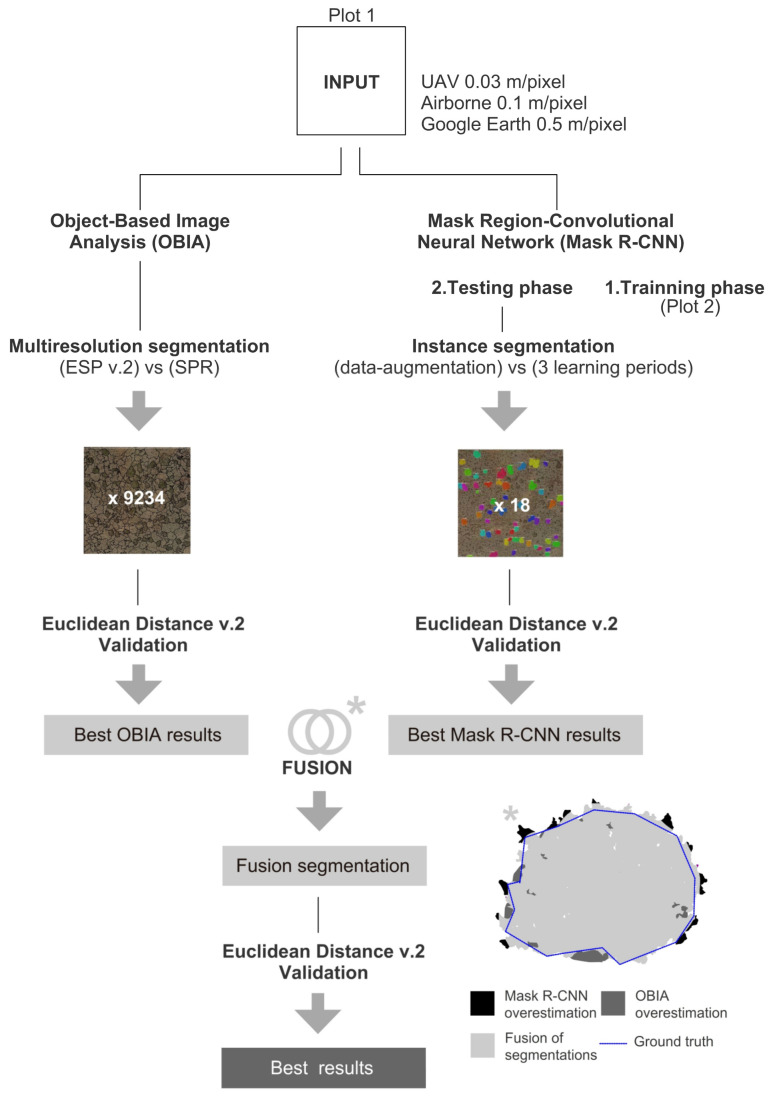
Workflow with the main processes carried out in this work. Asterisk shows an example of the result of the fusion of the segmentation results from OBIA and Mask R-CNN. OBIA: Object-Based Image Analysis; Mask R-CNN: Mask Region-based Convolutional Neural Networks; ESP v.2: Estimation of Scale Parameter v.2; SPR: Segmentation Parameters Range.

We set several experiments to assess the accuracy of the two different OBIA and Mask R-CNN segmenting scattered vegetation in drylands. We used the images of Plot 1 to test the OBIA and Mask R-CNN segmentation methods. The images of Plot 2 were used for the training phase in Mask R-CNN experiments exclusively (Figure 1). In Section 3.1, we describe OBIA experiments, focused on detecting the best parameters (i.e., Scale, Shape and Compactness) of a popularly used “multi-resolution” segmentation algorithm [71]. In Section 3.2. we described the Mask R-CNN experiments, in which we first evaluated the precision in the detection of shrubs (capture or notice the presence of shrubs) and second how accurate is the segmentation of those shrubs. Finally, in Section 3.3. we described the fusion of both methods and compared all the accuracies between them in Section 4.3.

### 3.1. OBIA Experiments

To obtain the optimal value of each parameter of the OBIA segmentation, we use two approaches:

(i) A ruleset called Segmentation Parameters Range (SPR) in eCognition v8.9 (Definiens, Munich, Germany) with the “multi-resolution” algorithm that segmented the images of Plot 1 by systematically increasing the Scale parameter in steps of 5 and the Shape and Compactness parameters in steps of 0.1. The Scale parameter ranged from 80 to 430, and the Shape and the Compactness from 0.1 to 0.9. We generated a total of 9234 results with possible segmentations of *Z. lotus* shrubs. The Scale parameter ranges were evaluated considering the minimum cover size (12 m^2^) and maximum cover size (311 m^2^) of the shrubs measured in the plot and the pixel size.
(ii) We also performed the semi-automatic method Estimation of Scale Parameter v.2 (ESP2; [70]) to select the best scale parameter. This tool performs semi-automatic segmentation of multiband images within a range of increasing Scale values (Levels), while the user previously defines the values of the Compactness and Shape parameters. Three options available in the ESP2 tool were tested: a) the hierarchical analysis Top-down (HT), starting from the highest level and segmenting these objects for lower levels; b) the hierarchical analysis Bottom-up (HB), which starts from the lower level and combines objects to get larger levels; and c) analysis without hierarchy (NH), where each scale parameter is generated independently, based only on the level of the pixel [64].

### 3.2. Mask R-CNN Experiments 

Mask R-CNN segmentation is divided in two phases: i) Training and ii) Testing phases. In the training phase, we selected 100 training polygons representing 100 shrub individuals with different sizes. The sampling was done using VGG Image Annotator [72] to generate a JSON file, which includes the coordinates of all the vertices of each segment, equivalent to the perimeter of each shrub. To increase the number of samples and reduce overfitting of the model, we applied data-augmentation and transfer-learning:

- Data augmentation aims to artificially increase the size of the dataset by slightly modifying the original images. We applied the filters of vertical and horizontal flip; Scale decrease and increase in the horizontal and vertical axis between 0.8 to 1.2; Rotation of 0 to 365 degrees; Shearing factor between −8 to 8; Contrast normalization with values of 0.75 and 1.5 per channel; Emboss with alpha 0, 0.1; Strength with 0 to 2.0; Multiply 0.5 and 1.5, per channel to change the brightness of the image (50–150% of the original value).
- Transfer-learning consists in using knowledge learnt from one problem to another related one [73], and we used it to improve the neural network. Since the first layers of a neural network extract low-level characteristics, such as colour and edges, they do not change significantly and can be used for other visual recognition works. As our new dataset was small, we applied fine adjustment to the last part of the network by updating the penultimate weights, so that the model was not overfitting, as mainly occurs between the first layers of the network. We specifically used transfer-learning on ResNet 101 [69] and used Region-based CNN with the pre-trained weights of the same architectures on COCO dataset (around 1.28 million images over 1000 generic object classes) [74].

We tested three different learning periods (100 steps per epoch) per model:

(A) 40 epochs with transfer-learning in heads,
(B) 80 epochs with 4 fist layers transfer-learning,
(C) 160 epochs with all layers transfer-learning.

We trained the algorithm based on the ResNet architecture with a depth of 101 layers with each of the three proposed spatial resolutions. We then evaluated the trained models in all possible combinations between the resolutions. We evaluated the use of data-augmentation and transfer-learning from more superficial layers to the whole architecture with different stages in the training process. Particularly:

(1.1) Trained with UAV images.
(1.2) Trained with UAV images and data-augmentation.
(2.1) Trained with airborne images.
(2.2) Trained with airborne images and with data-augmentation.
(3.1) Trained with Google Earth images.
(3.2) Trained with Google Earth images and data-augmentation.

We did the test phase using Plot 1. To identify the most accurate experiments, we evaluated the detection of the CNN-based models, and determined their Precision, Recall, and F1-measure [75] as:

(4) $$Precision\;=\;\frac{True\;Positives}{True\;Positives\;+\;False\;Positives},\;$$

(5) $$Recall\;=\;\frac{True\;Positives}{True\;positives\;+\;False\;Negatives},\;$$

(6) $$F1-measure\;=2\times \frac{Precision\;\times Recall}{Precision\;+\;Recall}\;$$

### 3.3. Fusion of OBIA and Mask R-CNN 

We combined the most accurate segmentations obtained using OBIA and Mask R-CNN, according to ED2 values (Figure 1). We let o_i_ denote the i-th OBIA polygon within the OBIA segmentation, O, and m_j_ denote the j-th Mask R-CNN polygon within the Mask R-CNN segmentation, C. Then we have O = {o_i_: i = 1, 2, ..., m} and C = {c_j_: j = 1, 2, ..., n}. Here, the subscripts i and j are sequential numbers for the polygons of the OBIA and Mask R-CNN segmentations, respectively. m and n indicate the total numbers of the objects segmented with OBIA and Mask R-CNN, respectively. m and n must be equal. Finally, the corresponding segment data sets extracted (Equation (7)) by the fusion are considered a consensus among the initially segmented objects as:

(7) $$OC_{ij}=\;areaO_{i}\;\cap \;areaC_{j}$$

where OC_ij_ is the intersected area between the segments of the OBIA segmentation (O_i_) and the area of the segments of the Mask R-CNN segmentation (C_j_). 

Finally, we estimate ED2 values of the final segmentation using validation shrubs from Plot 1, and we compared it with segmentation accuracy obtained by the different methods.

## 4. Results and Discussion

### 4.1. OBIA Segmentation 

**Figure 2:**
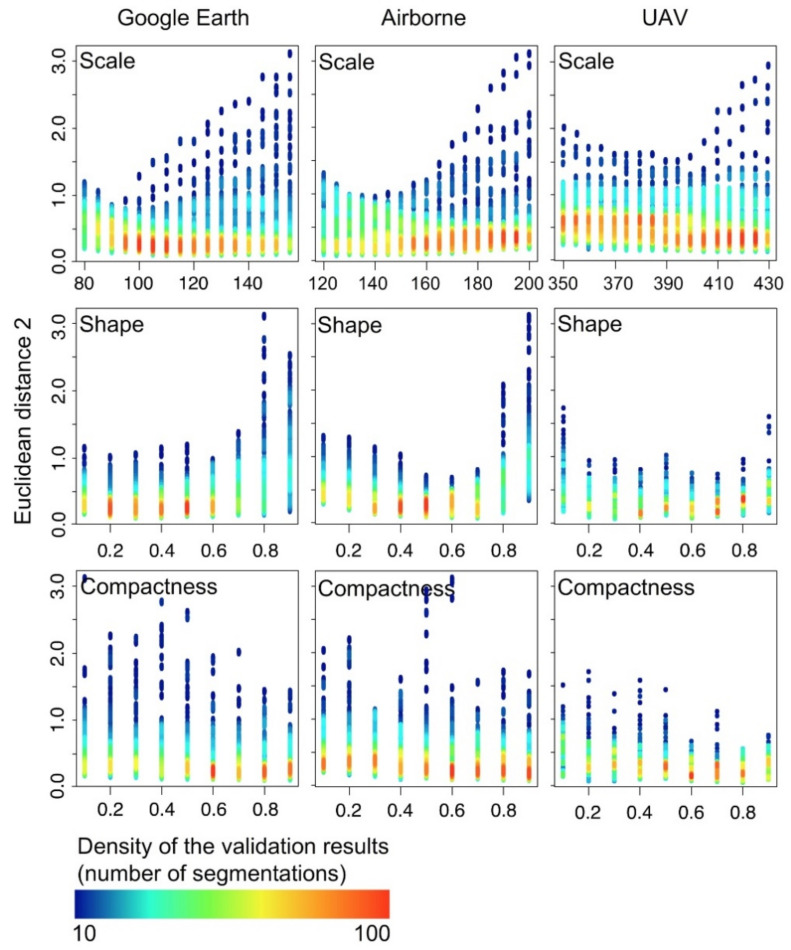
Relationship between Scale, Shape and Compactness parameters (X axis) evaluated using Euclidean distance v.2 (ED2; Y axis) in 9234 Object-based image analysis (OBIA) segmentations from Google Earth, Airborne and unmanned aerial vehicle (UAV) images. The rainbow palette shows the density of validation results. In red high density and in blue low density.

In total, 9234 segmentations were performed by SPR, 3078 for each image type (e.g., Google Earth, airborne and UAV). OBIA segmentation accuracy using the SPR presented large variability (Table 1), with values of ED2 ranging between 0.05 and 0.28. Segmentation accuracy increased with image spatial resolution. Thus, the higher the spatial resolution, the higher the Scale values and more accurate the segmentation was. This result was represented by a decrease in ED2 values of 0.14, 0.10 and 0.05 for Google Earth, airborne and UAV images, respectively. The best combinations of segmentation parameters along the different images were (Figure 2): (i) for the Google Earth image, Scale values ranging from 105 to 110, low Shape values of 0.3 and high Compactness values from 0.8 to 0.9; (ii) for the orthoimage from the airborne sensor, Scale values between 125 and 155, Shape of 0.6 and Compactness of 0.9; and (iii) for the UAV image, the optimal segmentation showed the highest Scale values, ranging from 360 to 420, whereas Shape and Compactness values were similar to the values of the Google Earth image. 

When we applied the semi-automatic method ESP2 to estimate the optimum value of the Scale parameter, we observed a similar pattern to that described for the SPR, with an increase in accuracy when increasing spatial resolution. The highest value of ED2 was for the Google Earth image segmentation results (ED2 = 0.25), decreasing for the orthoimage from the airborne sensor (ED2 = 0.15) and reaching the minimum value (best) in the UAV image (ED2 = 0.12). However, the results obtained by ESP2 were worse than the results obtained by the SPR method in all the images analysed (Table 1) with the largest differences in the image with the lowest spatial resolution (Google Earth). In the Google Earth images, the best method of analysis of the three options presented by the ESP2 tool was the hierarchical bottom level, with acceptable ED2 values, lower than 0.14 (Table 1). For the airborne images, the results were equal to Google Earth images (hierarchical bottom level). Conversely, the segmentation of the UAV image produced the best ED2 values when applying the ESP2 without hierarchical level. The computational time for the segmentation of the images was higher in ESP2 than SPR approach. In addition, the computation time of the analysis was also influenced by the number of pixels to analyse, it increased in higher spatial resolution images in computer with a Core i7-4790K, 4 GHz and 32G of RAM memory (Intel, Santa Clara, CA, USA) (Table 1).

### 4.2. Mask R-CNN Segmentation 

#### 4.2.1. Detection of Scattered Shrubs 

We obtained the best detection results for the models trained and evaluated with UAV images (F1-measure = 0.91) and the models trained with the highest number of epochs and data-augmentation activated (Table 2). The best transfer from a UAV trained model to a test with another resolution was to the image from the airborne sensor. Nevertheless, the Google Earth test image produced a similar result of F1-measure = 0.90. We consider that a model trained with data-augmentation and very high spatial resolution images (0.03 m/pixel) can generalize well to less accurate images such as those from Google Earth (0.5 m/pixel). Furthermore, when we trained the models with Google Earth images, we observed that it also generalised well to more precise resolutions (F1-measure = 0.90). For this reason, the detection of *Z. lotus* shrubs might be generalizable from any resolution less than 1 m/pixel.

#### 4.2.2. Segmentation Accuracy for Detected Shrubs 

**Figure 3:**
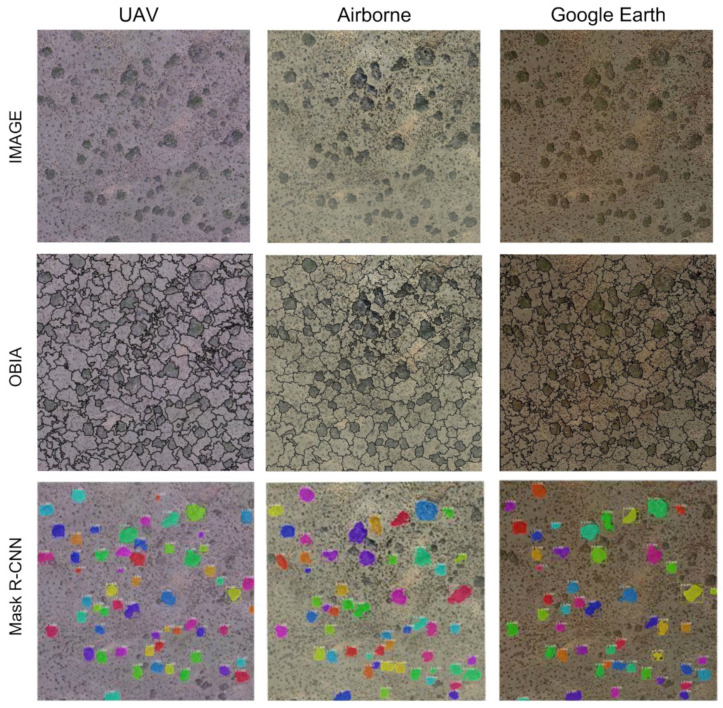
Examples of segmentation of images from Plot 1 using Object-based Image Analysis (OBIA; **Top**) and Mask Region-based Convolutional Neural Networks (Mask R-CNN; **Down**) on Google Earth, Airborne and Unmanned Aerial Vehicle (UAV) images. The different colours in the Mask R-CNN approach are to differentiate the shrubs individually.

The best segmentation accuracy was obtained with the models trained and tested with the same source of images, reaching values of ED2 = 0.07 in Google Earth ones. However, when the model trained with Google Earth images was tested in a UAV image, the ED2 resulted in 0.08. Moreover, the effect of data-augmentation was counterproductive in models trained with airborne images and only lowered ED2 (best results) in models trained with the UAV image. In general, data-augmentation helped to generalise between images but did not obtain a considerable increase in precision in models trained and tested with the same image resolution (Table 3 and Figure 3).

### 4.3. Fusion of OBIA and Mask R-CNN 

Our results showed that the fusion between OBIA and Mask R-CNN methods in very high-resolution RGB images is a powerful tool for mapping scattered shrubs in drylands. We found that the individual segmentations by using OBIA and Mask R-CNN independently were worse than the fusion of both. The accuracy of the fusion of OBIA and Mask R-CNN was higher than the accuracies of the separate segmentations (Table 4), being the most accurate segmentation of all the experiments tested in this work, with an ED2 = 0.038. However, the fusion between results on Google Earth images only improved the ED2 by 0.02. Therefore, the fusion of both segmentation methods provided the best segmentation over the previous methods (OBIA (ED2 = 0.05) and Mask R-CNN (ED2 = 0.07)), in very high-resolution images to segment scattered vegetation in drylands. Moreover, by merging the results of both methodologies (OBIA ∩ Mask R-CNN), the accuracy increases with an ED2 = 0.03. 

To our knowledge, the effect of mixing these two methodologies has not been studied until the date, and it might be vital to improving future segmentation methods. As can be seen in the conceptual framework (Figure 1), it is reasonable to think that the higher the resolution and, therefore, the higher the detail at the edges of vegetation represented in the images, the fusion will improve the final precision of the segmentation. Nevertheless, in images with lower resolution, the fusion improved but to a minor degree. 

The spatial resolution of the images affected the accuracy of the segmentation, providing outstanding results in all segmentation methods and spatial resolutions. However, according to [57], we observed that the spatial resolution and Scale parameter played a key role during the segmentation process and controlled the accuracy of the final segmentations. In non-fusion segmentation methods (OBIA or Mask R-CNN) the segmentation accuracy was higher in the spatial resolution image from UAV and OBIA up to ED2 = 0.05. However, when the object to be segmented is larger than the pixel size of the image, the spatial resolution of the image is of secondary importance [37,57,76,77]. For this reason, as the scattered vegetation in this area presents a mean size of 100 m^2^ [5], corresponding to 400 pixels of Google Earth image, only slight increases in segmentation accuracy were observed as the spatial resolution increased. Moreover, the overestimation of the area of each shrub was not significant as the images spatial resolution increased. Therefore, Google Earth images could be used to map scattered vegetation in drylands, if the plants to be mapped are larger than the pixel size. This result opens a wide range of new opportunities for vegetation mapping in remote areas where UAV or airborne image acquisition is difficult or acquiring commercial imagery of very high-resolution is very expensive. These results are promising and highlight the usefulness of free available Google Earth images for big shrubs mapping with only a negligible decrease in segmentation accuracy when compared with commercial UAV or airborne images. However, the segmentation of vegetation could be better if we use the near infrared NIR band since vegetation highlights in this range of the spectrum (e.g., 750 to 2500 nm) or used in vegetation indices such as the normalized difference vegetation index (NDVI) or Enhanced vegetation index (EVI). Finally, very high spatial resolution UAV images need much more computational time and are expensive and not always possible to obtain at larger scales in remote areas, hampering their use.

## 5. Conclusions

Our results showed that both OBIA and Mask R-CNN methods are powerful tools for mapping scattered vegetation in drylands. However, both methods were affected by the spatial resolution of the orthoimages utilized. We have shown for the first time that the fusion of the results from these methods increases, even more, the precision of the segmentation. This methodology should be tested on other types of vegetation or objects in order to prove to be fully effective. We propose an approach that offers a new way of fusing these methodologies to increase accuracy in the segmentation of scattered shrubs and should be evaluated on other objects in very high-resolution and hyperspectral images. 

Using images with very high spatial resolution could provide the required precision to further develop methodologies to evaluate the spatial distribution of shrubs and dynamics of plant populations in global drylands, especially when utilizing free-to-use images, like the ones obtained from Google Earth. Such evaluation is of particular importance in drylands of developing countries, which are particularly sensitive to anthropogenic and climatic disturbances and may not have enough resources to acquire airborne or UAV imagery. For these reasons, future methodologies as the one presented in this work should focus on using freely available datasets.

In this context, the fusion of OBIA and Mask R-CNN could be extended to a larger number of classes of shrub and tree species or improved with the inclusion of more spectral and temporal information. Furthermore, this approach could improve the segmentation and monitoring of the crown of trees and arborescent shrubs in general, which are of particular importance for biodiversity conservation and for reducing uncertainties in carbon storages worldwide [78]. Recently, scattered trees have been identified as key structures for maintaining ecosystem services provision and high levels of biodiversity [43]. Global initiatives could benefit largely from CNNs, including those recently developed by FAO [79] to provide the forest extent in drylands. The uncertainties in this initiative [80,81] might be reduced implementing our approach CNN-based to segment trees. Tree and shrub segmentation methods could provide a global characterization of forest ecosystem structures and population abundances as part of the critical biodiversity variables initiative [82,83]. In long-lived shrubs where the precision of the segmentation is key for monitoring the detection of disturbances (e.g., pests, soil loss or seawater intrusion) [5]. Finally, the monitoring of persistent vegetation with minimal cover changes over decades could benefit from fusion approaches in the segmentation methods proposed. 

## Figures and Tables

**Table 1:** Segmentation accuracies of Object-Based Image Analysis (OBIA) among the three spatial resolutions evaluated. For each segmentation type, only the most accurate combination of Scale, Shape, and Compactness is shown. ESP2/HB: Estimate Scale Parameter v.2 (ESP2) with Bottom-up Hierarchy; ESP2/HT: ESP2 with Top-down Hierarchy; ESP2/NH: ESP2 Non-Hierarchical; SPR: Segmentation with Parameters Range. Closer values to 0 indicate accurate segmentations. In bold the most accurate results.

			Segmentation Parameters			Segmentation Quality	
Image Source	Resolution (m/Pixel)	Segmentation Method	Scale	Shape	Compactness	ED2	Average Time (s)
Google Earth	0.5	ESP2/HB	100	0.6	0.9	0.25	365
		ESP2/HT	105	0.7	0.5	0.26	414
		ESP2/NH	105	0.5	0.1	0.28	2057
		**SPR**	**90**	**0.3**	**0.8**	**0.2**	**18**
Airborne	0.1	ESP2/HB	170	0.5	0.9	0.14	416
		ESP2/HT	160	0.5	0.9	0.15	650
		ESP2/NH	160	0.5	0.5	0.14	3125
		**SPR**	**155**	**0.6**	**0.9**	**0.1**	**24**
UAV	0.03	ESP2/HB	355	0.3	0.7	0.12	5537
		ESP2/HT	370	0.5	0.7	0.11	8365
		ESP2/NH	350	0.5	0.7	0.1	40,735
		**SPR**	**420**	**0.1**	**0.8**	**0.05**	**298**

**Table 2:** Test results of Mask Region-based Convolutional Neural Networks (Mask R-CNN) experiments in three different spatial resolutions images. TP: True Positive; FP: False Negative; FN: False Negative. Precision, Recall, and F1-measure were used for detection results. In bold the most accurate results.

Experiments/Image		TP	FP	FN	Precision	Recall	F1
1.1.A	UAV	55	5	10	0.92	0.85	0.88
	Airborne	56	4	9	0.93	0.86	0.90
	GE	50	1	15	0.98	0.77	0.86
1.1.B	UAV	59	6	6	0.91	0.91	0.91
	Airborne	60	7	5	0.90	0.92	0.91
	GE	55	2	10	0.96	0.85	0.90
1.1.C	**UAV**	**55**	**1**	**10**	**0.98**	**0.85**	**0.91**
	Airborne	52	3	13	0.94	0.80	0.87
	GE	53	0	12	1	0.81	0.89
1.2.A	UAV	53	1	12	0.98	0.82	0.89
	Airborne	54	1	11	0.98	0.83	0.90
	GE	42	3	23	0.93	0.65	0.76
1.2.B	UAV	55	1	10	0.98	0.85	0.91
	Airborne	50	2	15	0.96	0.77	0.85
	GE	50	2	15	0.96	0.77	0.85
1.2.C	**UAV**	**56**	**3**	**8**	**0.95**	**0.87**	**0.91**
	Airborne	52	3	13	0.94	0.80	0.87
	GE	54	1	12	0.98	0.81	0.89
2.1.A	UAV	41	0	24	1	0.63	0.77
	Airborne	38	0	27	1	0.58	0.74
	GE	34	1	31	0.97	0.52	0.68
2.1.B	UAV	47	0	18	1	0.72	0.84
	**Airborne**	**55**	**3**	**10**	**0.95**	**0.85**	**0.89**
	GE	50	1	16	0.98	0.76	0.85
2.1.C	UAV	52	1	13	0.98	0.80	0.88
	Airborne	58	3	7	0.95	0.88	0.91
	GE	54	1	12	0.98	0.82	0.89
2.2.A	UAV	31	0	34	1	0.48	0.65
	Airborne	48	1	17	0.98	0.74	0.84
	GE	38	1	27	0.97	0.58	0.73
2.2.B	UAV	38	1	27	0.97	0.58	0.73
	Airborne	46	1	19	0.98	0.71	0.82
	GE	47	3	18	0.94	0.72	0.82
2.2.C	UAV	46	1	19	0.98	0.70	0.82
	**Airborne**	**51**	**2**	**14**	**0.96**	**0.78**	**0.86**
	GE	50	2	15	0.96	0.77	0.85
3.1.A	UAV	37	0	28	1	0.57	0.73
	Airborne	43	0	22	1	0.66	0.80
	GE	41	1	24	0.98	0.63	0.77
3.1.B	UAV	48	1	17	0.98	0.74	0.84
	Airborne	51	1	14	0.98	0.78	0.87
	**GE**	**54**	**1**	**11**	**0.98**	**0.83**	**0.90**
3.1.C	UAV	52	1	13	0.98	0.80	0.88
	Airborne	52	1	13	0.98	0.80	0.88
	GE	54	2	11	0.96	0.83	0.89
3.2.A	UAV	54	1	11	0.98	0.83	0.90
	Airborne	56	4	9	0.93	0.86	0.90
	GE	53	2	12	0.96	0.82	0.88
3.2.B	**UAV**	**56**	**3**	**9**	**0.95**	**0.86**	**0.90**
	Airborne	54	5	11	0.92	0.83	0.87
	GE	53	3	12	0.95	0.82	0.88
3.2.C	UAV	54	3	11	0.95	0.83	0.89
	Airborne	52	3	13	0.95	0.80	0.87
	GE	52	3	13	0.95	0.80	0.87

**Table 3:** Segmentation accuracies of Mask Region-based Convolutional Neural Networks (Mask R-CNN). PSE: Potential Segmentation Error; NSR: Number Segmentation Ratio; ED2: Euclidean Distance v.2. In bold the most accurate results.

Best Experiment	Image Train	Image Test	PSE	NSR	ED2
1.1.C	UAV	UAV	0.0532	0.1290	0.1396
1.2.C	UAV	UAV	0.0512	0.0967	0.1095
**2.1.C**	**Airborne**	**Airborne**	**0.0408**	**0.0645**	**0.0763**
2.2.C	Airborne	Airborne	0.0589	0.0645	0.0873
**3.1.B**	**GE**	**GE**	**0.0414**	**0.0645**	**0.0767**
**3.2.B**	**GE**	**UAV**	**0.0501**	**0.0645**	**0.0816**

**Table 4:** Segmentation accuracies of the fusion of Object-Based Image Analysis (OBIA) and Mask Region-based Convolutional Neural Networks (Mask R-CNN). PSE: Potential Segmentation Error; NSR: Number Segmentation Ratio; ED2: Euclidean Distance v.2. In bold the most accurate results.

Best Experiment	Best OBIA (ED2)	Best Mask R-CNN (ED2)	PSE	NSR	ED2
**1.1.C**	**0.05**	**0.13**	**0.02**	**0.03**	**0.0386**
1.2.C	0.05	0.10	0.02	0.03	0.0417
2.1.C	0.10	0.07	0.02	0.03	0.0388
2.2.C	0.10	0.08	0.05	0.06	0.0395
3.1.B	0.20	0.07	0.00	0.06	0.0645
3.2.B	0.20	0.08	0.00	0.06	0.0645

## Data Availability

All drone and airborne orthomosaic data, shapefile and code will be made available on request to the correspondent author’s email with appropriate justification.

## Abbreviations

The following abbreviations are used in this manuscript:

**Abbreviation**	**Description**
CCD	Charge-Coupled Device
ED2	Euclidean Distance v.2
ESP2	Estimation Scale Parameter v.2
ETRS	European Terrestrial Reference System
HB	Bottom-up Hierarchy
HT	Top-down Hierarchy
JSON	JavaScript Object Notation
NH	Non-Hierarchical
NSR	Number-of-Segmentation Ratio
OBIA	Object-based Image Analysis
R-CNN	Region—Convolutional Neural Networks
RGB	Red Green Blue
SPR	Segmentation Parameters Range
UAV	Unmanned aerial vehicle
UTM	Universal Transverse Mercator
VGG	Visual Geometry Group
